# Two-Month Duration of Esophageal Button Battery Impaction in a 23-Month-Old Boy: A Case Report

**DOI:** 10.7759/cureus.74134

**Published:** 2024-11-21

**Authors:** Abdulrahman Abaalkhail, Yousef Alshehri, Abdullah Almutairi, Abdulaziz Ajeebi, Msaed Alotaibi, Ziyad Almutairi

**Affiliations:** 1 Department of Pediatrics Emergency, King Abdullah Specialized Children’s Hospital, Ministry of the National Guard Health Affairs, Riyadh, SAU; 2 Department of Pediatrics, King Abdullah Specialized Children’s Hospital, Ministry of the National Guard Health Affairs, Riyadh, SAU; 3 College of Medicine, King Saud Bin Abdulaziz University for Health Sciences, Riyadh, SAU

**Keywords:** button batteries, case report, esophageal impaction, foreign body ingestion, pediatric emergency

## Abstract

Ingestion of foreign bodies, particularly button batteries (BB), is a common pediatric emergency. Esophageal impaction of BB is associated with life-threatening complications. Damage can be manifested within hours from ingestion; therefore, immediate removal is recommended. A 23-month-old boy presented to the emergency department following an incidental chest X-ray that revealed a BB lodged in the upper esophagus, initially overlooked during a prior visit. The family reported that the child had been experiencing persistent cough, intermittent fever, vomiting, and poor oral intake over the last two months. Upon admission, the child was stable, but laboratory tests indicated elevated inflammatory markers. A multidisciplinary team was promptly assembled for management, leading to a successful extraction of the BB in the operating room. Postoperatively, the child developed secondary hemophagocytic lymphohistiocytosis and methicillin-sensitive Staphylococcus aureus infection, which were effectively treated. After discharge, the patient was followed up in the clinic, and no complications were observed. Although BB ingestions are not the commonest among all types of foreign body ingestion, their serious complications have been evidence-based published.

## Introduction

Ingestion of foreign bodies is a common case presentation in pediatric emergency medicine (PEM) [1]. Coins, button batteries (BB), and toys represent the most common types of foreign body ingestions among the pediatric age group, particularly children below the age of five years [2,3]. Recently, BB ingestions have been encountered more frequently in PEM as demonstrated in the literature, the proposed explanation attributed to the trend of using electrical devices [4,5]. Fortunately, many BB ingestions pass throughout gastrointestinal tract without causing harm; however, some have a potential complication and eventually can lead to death [1,3]. Severe complications are associated with many factors. The anatomical lodgement of ingested BB, its size, and the age group was all found to be relatively significant to determine the morbidity and mortality [6-8]. Esophageal lodgement of BB is associated with serious outcomes, such as perforation, hemorrhage, fistula, vocal cord paralysis, and death [8,9]. Therefore, the North American Society for Pediatric Gastroenterology, Hepatology, and Nutrition (NASPGHAN) recommended immediate removal of BB lodged in esophagus because the damage occurs within two hours from ingestion [10]. This case presents a child with a persistent cough for a duration of two months, found incidentally to have BB in his esophagus.

## Case presentation

We present here the case of a 23-month-old boy, previously healthy and vaccinated up to his age, brought to the emergency department by his parents following an incidental finding of a BB in his esophagus reported on a chest X-ray, which was done in another healthcare facility. The family reported that the child had been experiencing persistent cough, intermittent fever, vomiting, and poor oral intake for the past two months. The mother recounted an episode of unwitnessed BB ingestion two months earlier, initially missed during a prior visit to a healthcare facility, where the child was discharged with a diagnosis of constipation after an abdominal KUB X-ray without capturing the chest.

**Figure 1:**
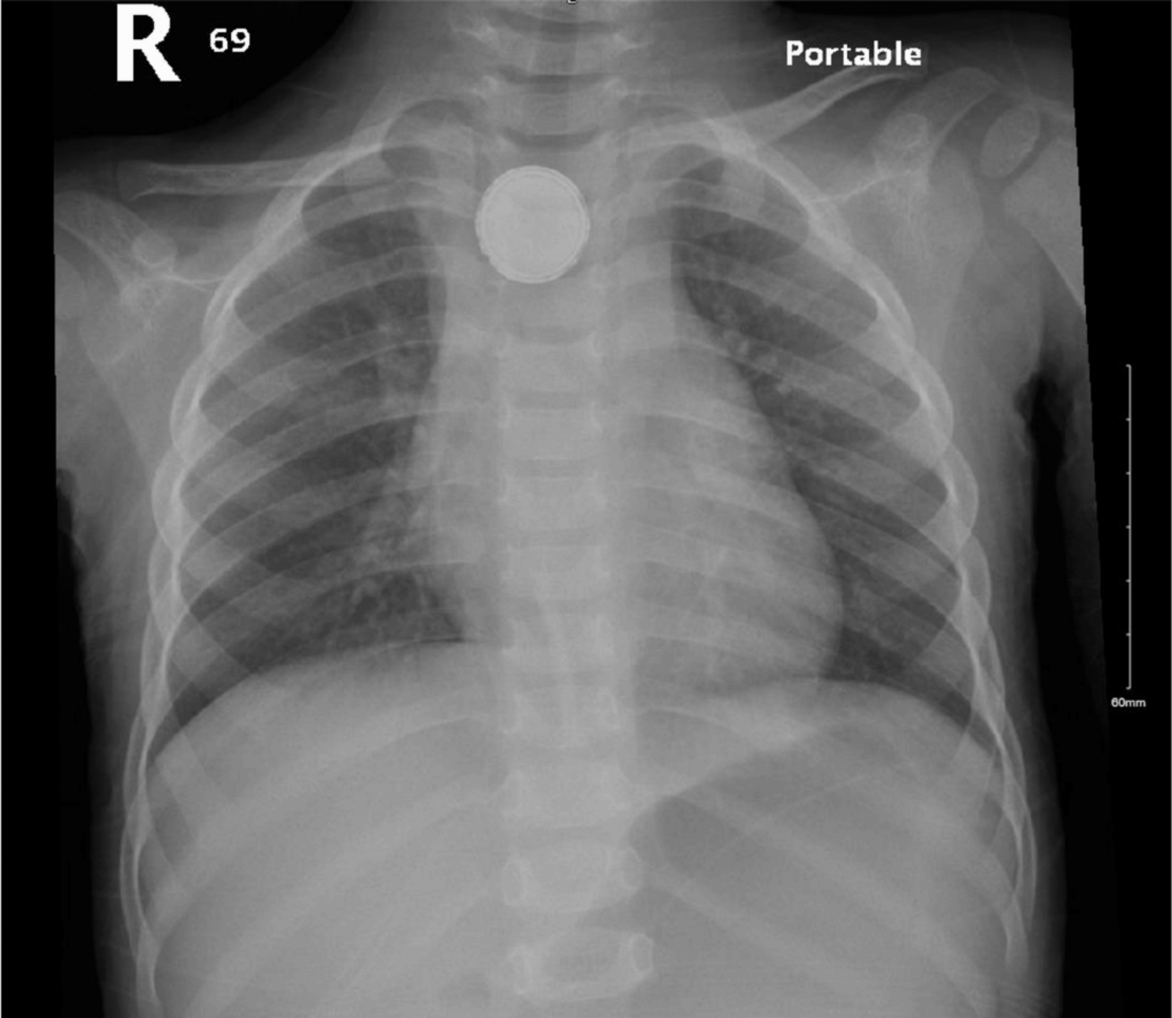
Chest X-ray showed rounded metallic density with double-density edges suggestive of a button battery in the upper esophagus.

Upon presentation to the emergency department, the child was found to be vitally stable, with no signs of respiratory distress, cyanosis, or abnormal movements. Physical examination revealed an active, alert, and well-hydrated child. His chest examination showed clear lung fields, and his abdomen was soft, lax, and non-tender. Laboratory investigations showed an elevated white blood cell count of 25 × 10⁹/L, with neutrophils at 17 × 10⁹/L. Inflammatory markers were also slightly elevated, with an ESR of 16 mm/hour and CRP of 32 mg/L (Table 1). A chest X-ray confirmed the presence of a BB in the upper part of the esophagus (Figure 1).

**Table 1:** Laboratory results.

Exam. Name		Reference Value
Baso #	0.05	0-0.1 x 10^9^/L
Baso %	0.19	~%
Eos #	0.01	0.1-0.7 x 10^9^/L
Eos %	0.02	~%
Neut %	69.6	~%
Lymph %	22.2	~%
Mono #	1.82	0.1-1.1 x 10^9^/L
Mono %	7.26	~%
Neut #	17.4	0.8-5.4 x 10^9^/L
Lymph #	5.55	1.4-8.4 x 10^9^/L
Hgb	99	110-145 gm/L
WBC	7.17	4-12 x 10^9^/L
RBC	3.93	3.9-5.6 x 10^12^/L
Hct	0.287	0.31-0.45 L/L
MCV	72.9	75-89 fL
MCH	25.2	25-30 pg
MCHC	346	315-350 g/L
RDW	15.8	11.5-14.5%
NRBC#	0	0-3 x 10^9^/L
Platelet	254	150-450 x 10^9^/L
NRBC %	0	0-5%
MPV	8.8	7.4-10.4 fL
ESR	16	0-15 mm/hour
CRP	32	~5 mg/L

**Figure 2:**
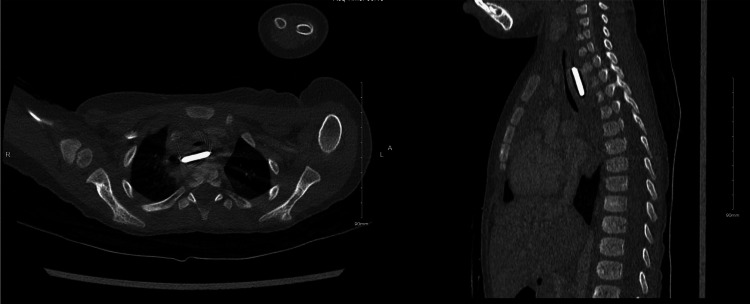
Chest computed tomography showed an impacted button battery in the upper thoracic of the esophagus.

Given the concerning history of a long-standing esophageal foreign body ingestion and impaction, a multidisciplinary team, including pediatric gastroenterology, pediatric surgery, pediatric ENT, and cardiac surgery, was promptly consulted. The child was admitted to the pediatric intensive care unit (PICU) for close observation, and a chest CT angiography was performed to assess for potential complications such as esophageal perforation or fistula formation. The chest CT angiography scan confirmed the presence of an impacted BB in the upper thoracic esophagus causing moderate tracheal narrowing, and Focal right esophageal air-filled outpouching, highly concerning for sealed perforation. The scan showed no definite sign of vascular injury (Figure 2).

On day two of the hospital stay, the multidisciplinary team meeting was held on the same day as the operating room (OR) and before the procedure, the intervention steps were reviewed and finalized among the different teams, anticipation for possible complications was addressed, and the next step if any complication occurred was discussed and planned clearly. The decision was made to remove the BB in the cardiac OR under the supervision of the multidisciplinary team. During the procedure, the BB was successfully removed without any complications. Postoperatively, an upper endoscopy was performed, revealing esophageal granulation tissue and necrotic areas at 10 cm from the incisor, but no circumferential burn or apparent fistula was identified. The child remained stable throughout the procedure and recovery.

Following the surgery, the child was kept nil per os (NPO) and received total parenteral nutrition (TPN) through a peripherally inserted central catheter (PICC) line. Two weeks after the removal of the BB, he developed a fever, with a nasal pharyngeal aspirate (NPA) returning positive for human rhinovirus. Laboratory investigations showed elevated ferritin and other markers suggestive of hemophagocytic lymphohistiocytosis (HLH), a diagnosis of secondary HLH was made. The child was started on intravenous (IV) pulse methylprednisolone, followed by oral prednisolone with a tapering dose. All HLH markers improved and normalized during the course of treatment.

Three weeks after the procedure, the child developed another spike of fever, and blood cultures were positive for methicillin-sensitive Staphylococcus aureus (MSSA). He was initially treated with IV vancomycin and, later, with the recommendations from the ID team, switched to IV cefazolin for a total of 14 days. Two subsequent blood cultures were negative, and the PICC line was removed by the patient during the hospital stay.

On day 21 of admission, and after a negative upper gastrointestinal series (UGIS), the child was gradually reintroduced to a liquid diet, which he tolerated well, followed by a soft diet without any signs of aspiration or clinical deterioration.

The child was kept in the hospital for a total of 45 days and was discharged in stable condition, clinically well, afebrile, and tolerating an oral diet. His physical examination upon discharge was unremarkable, with normal vital signs and no evidence of ongoing esophageal injury or systemic infection. The patient’s post-discharge course was uneventful, and he continues to be followed up by the relevant specialties to monitor for any potential late complications.

## Discussion

Foreign body ingestions in children have been a challenging presentation at the emergency department ED. BB ingestion incidence has been noticed to have increased recently, according to Thabet et al., and this increase in battery ingestion is apparently secondary to the global daily usage of electrical devices [4]. Damage of BB thought to be achieved through different mechanisms has been discussed in previous published studies. These are the three main mechanisms: electrical damage that is generated by the battery pole against tissue, mechanical damage causing necrosis by the direct pressure of the battery, or chemical damage when leakage of the battery contents exerts corrosive damage to the tissue wall [4,10,11].

Clinical presentation varies with a wide spectrum from being totally asymptomatic to severely sick patients. Respiratory and gastrointestinal symptoms, such as coughing, stridor, vomiting, dehydration, lethargy, drooling, dysphagia, chest pain, and fever, are the most frequent complaints in ED settings [1,4,7]. In line with our case, the patient has had persistent cough, vomiting, and fever since the time of ingestion.

The size of the BB has been found in the literature to be significantly associated with esophageal impaction. The anatomy of the esophagus increases the chance of BB lodgment, as it has an area of narrowing with weak smooth muscle peristalsis [3,10]. Logically, the chance of esophageal impaction increases in children younger than five years old and ingestion BB more than 20 mm in diameter [7,8].

Although it is a challenge for physicians to diagnose a child with foreign body ingestion, suspicion has to be raised for BB ingestions based on the clinical picture along with detailed history taking [12]. According to Shew et al., 60% of the patients with esophageal impaction of BB are symptomatic within 24 hours from ingestion and 80% within the first week [13]. Therefore, for children who are symptomatic, simple imaging of the chest can be done to differentiate BB from other foreign bodies, as BB has a unique double-rimmed appearance on X-ray.

Lodgment of BB in the esophagus prompts the removal of BB as soon as possible to prevent further complications that can be manifested as early as a few hours from ingestion [3,10,14]. Delaying the removal of the esophageal impaction of BB is associated with life-threatening serious outcomes [3]. The most frequent complications are perforation, hemorrhage, fistula, vocal cord paralysis, and death [8].

Complications of BB ingestion can be seen even after days of removal [6,15]. Previous reports stated that trachea-esophageal fistula was manifested after six days of removal. Moreover, hemorrhage was found to be reported even after 18 days of removal [16]. Unlike our case, Guinet et al. published a case study for a child whose age is 17 months, she had esophageal BB ingestion for 19 days, and the patient passed away secondary to esophageal rapture and aspiration of the blood [17].

A similar case was published in 2016 by Marshalla et al. wherein a 15-month-old girl presented to the ED with a three-month history of refusal to eat food and was found to have BB lodged in her esophagus. Removal was done with no complications mentioned. The patient was discharged on day two post-operatively, and a follow-up after one month showed a normal child with normal eating habits [3].

## Conclusions

Patients with esophageal impaction of BB might present initially with mild symptoms or even be asymptomatic. Early recognition of such a presentation is crucially demanded by clinicians to prevent life-threatening complications, poor outcomes, and mortality. The prolonged duration of esophageal impaction is directly proportional to further tissue damage; however, rare cases have been found to have BB ingestions with minor or no complications being reported.
